# CircPVT1 promotes migration and invasion by regulating miR‐490‐5p/ *HAVCR2* axis in osteosarcoma cells

**DOI:** 10.1111/jcmm.18269

**Published:** 2024-04-03

**Authors:** Chunbin Zhou, Lois Balmer, Manshu Song, Kezhou Wu, Wei Wang, Hu Wang

**Affiliations:** ^1^ Department of Orthopaedics First Affiliated Hospital of Shantou University Medical College Shantou Guangdong China; ^2^ Center for Precision Health, School of Medical and Health Science Edith Cowan University Perth Western Australia Australia; ^3^ First Affiliated Hospital of Shantou University Medical College Shantou Guangdong China; ^4^ Minimally Invasive Spine Center First Affiliated Hospital of Shantou University Medical College Shantou Guangdong China

**Keywords:** circPVT1, *HAVCR2*, invasion, migration, miR‐490‐5p, osteosarcoma

## Abstract

Circular RNAs (circRNAs) play an important role in the progression of osteosarcoma. However, the precise function of circPVT1 in osteosarcoma remains elusive. This study aims to explore the molecular mechanism underlying the involvement of circPVT1 in osteosarcoma cells. We quantified circPVT1 expression using qRT‐PCR in both control and osteosarcoma cell lines. To investigate the roles of circPVT1, miR‐490‐5p and *HAVCR2* in vitro, we separately conducted overexpression and inhibition experiments for circPVT1, miR‐490‐5p and *HAVCR2* in HOS and U2OS cells. Cell migration was assessed through wound healing and transwell migration assays, and invasion was measured via the Matrigel invasion assay. To elucidate the regulatory mechanism of circPVT1 in osteosarcoma, a comprehensive approach was employed, including fluorescence in situ hybridization, qRT‐PCR, Western blot, bioinformatics, dual‐luciferase reporter assay and rescue assay. CircPVT1 expression in osteosarcoma cell lines surpassed that in control cells. The depletion of circPVT1 resulted in a notable reduction in the in vitro migration and invasion of osteosarcoma cells. Mechanism experiments revealed that circPVT1 functioned as a miR‐490‐5p sequester, and directly targeted *HAVCR2*. Overexpression of miR‐490‐5p led to a significant attenuation of migration and invasion of osteosarcoma cells, whereas *HAVCR2* overexpression had the opposite effect, promoting these abilities. Additionally, circPVT1 upregulated *HAVCR2* expression via sequestering miR‐490‐5p, thereby orchestrating the migration and invasion in osteosarcoma cells. CircPVT1 orchestrates osteosarcoma migration and invasion by regulating the miR‐490‐5p/*HAVCR2* axis, underscoring its potential as a promising therapeutic target for osteosarcoma.

## 1 INTRODUCTION

Osteosarcoma (OS) is a relatively uncommon but highly aggressive primary bone cancer that primarily affects young children and adolescents, with an incidence ranging from two to five cases per million.^1^ OS predominantly manifests in the long bones, but can potentially develop in any bone throughout the body.^2^ Since the implementation of adjuvant chemotherapy in the 1980s has led to a 5‐year survival rate of up to 60% for OS patients with localized lesions, the prognosis for metastatic OS patients remains around 20%.^3^, ^4^ Consequently, delving into the mechanisms underlying OS metastasis is imperative to identify precisive potential therapeutic targets.

Circular RNAs (circRNAs) represent a class of non‐coding RNAs characterized by a closed‐loop structure formed through a back‐splicing process.^5^ Recently, circRNAs have been found to have remarkable stability, tissue and cell specificity, and play an essential role in various diseases.^6^ Furthermore, accumulating evidence highlights the involvement of circRNAs in the progression of OS.^7^ While the functions of most circRNAs remain largely unknown, one established role attributed to circRNAs is their ability to function as competing endogenous RNAs (ceRNAs), by sequestering miRNAs through their binding sites, and consequently influencing the expression of miRNA target genes.^8^ For example, upregulated hsa_circ_0006990 facilitates the growth of small cell cancer by acting as a positive regulator of *IGF1R* via sequestering miR‐377‐3p and miR‐494‐3p.^9^

In the context of OS, numerous circRNAs have been reported to actively partake in the regulation of cancer cells malignant traits.^10^, ^11^, ^12^ Hence, these findings support the significance and importance of exploring the roles that circRNAs play in the regulatory mechanism governing the progression of OS.

The circRNA hsa_circ_0001821 also known as circPVT1, originates from exon 2 (410 nt) of plasmacytoma variant translocation 1 (*PVT1*) gene (8q24), a widely recognized region associated with cancer risk.^13^ Previously, circPVT1 was identified as being upregulated in OS and associated with poor prognosis.^14^ Additionally, circPVT1 has been shown to play a pivotal role in promoting the proliferation, migration, invasion and chemotherapy resistance of OS cells by functioning as a ceRNA.^15^, ^16^, ^17^

However, the precise regulatory mechanisms through which circPVT1 operates in OS requires further investigation. Consequently, our study endeavoured to understand the impact and regulatory mechanism of circPVT1 on the migration and invasion of OS cells. This research aimed to contribute additional insight into the role played by circPVT1 in the progression of OS.

## 2 MATERIALS AND METHODS

### 2.1 Cell culture

A normal osteoblast cell line (hFOB1.19) was cultured in Dulbecco's modified Eagle's medium (DMEM)/Nutrient Mixture F12 supplemented with 0.3 mg/mL G418, 10% fetal bovine serum (FBS) and 1% penicillin/streptomycin (Procell, China). The OS cell lines MG63 and HOS were cultured in DMEM (Gibco, USA), while U2OS was cultured in McCOY's5A medium, all medium were supplemented with 10% FBS (Gibco, USA) and 1% penicillin/streptomycin. All cells were cultured at 37°C incubator containing 5% CO_2_.

### 2.2 Cell transfection

CircPVT1 was cloned into the pcDNA3.1 (+) vector (GenePharma, China) for overexpression studies. *HAVCR2* (hepatitis A virus cellular receptor 2) was cloned into pEX‐5 vector (GenePharma, China) for overexpression. The empty vector was used as a negative control (NC). The transfection of vectors was performed using Lipofectamine 3000 (Invitrogen, USA) at 37°C for 48 h. Small interfering RNAs (siRNAs) against circPVT1 (si‐circPVT1) and *HAVCR2* (si‐*HAVCR2*), and siRNA NC (si‐NC) were purchased from Sangon Biotech, China. miR‐490‐5p mimics, inhibitor, NC and NC inhibitor were synthesized by GenePharma, China. The transfection of oligonucleotides (siRNAs, miRNA mimics, inhibitor and corresponding NCs) was conducted using RNATransMate (Sangon Biotech, China) at 37°C for 48 h. All oligonucleotide sequences are shown in Table 1.

**TABLE 1:** Sequence of oligonucleotides for cell transfection.

Name		Sequence
circPVT1 siRNA	Sense	CUGUCAGCUGCAUGGAGCUUCGU
	Anti‐sense	ACGAAGCUCCAUGCAGCUGACAG
*HAVCR2* siRNA	Sense	CUCAGGACUGAUGAAAGGGAUTT
	Anti‐sense	AUCCCUUUCAUCAGUCCUGAGTT
siRNA NC	Sense	UUCUCCGAACGUGUCACGUTT
	Anti‐sense	ACGUGACACGUUCGGAGAATT
miR‐490‐5p mimics	Sense	CCAUGGAUCUCCAGGUGGGU
	Anti‐sense	CCACCUGGAGAUCCAUGGUU
mimics NC	Sense	UUCUCCGAACGUGUCACGUTT
	Anti‐sense	ACGUGACACGUUCGGAGAATT
miR‐490‐5p inhibitor	Sense	ACCCACCUGGAGAUCCAUGG
inhibitor NC	Sense	CAGUACUUUUGUGUAGUACAA

*Note*: All sequences are written in 5′–3′ direction.

### 2.3 RNA extraction and quantitative real‐time polymerase chain reaction (qRT‐PCR)

RNA was extracted using TRNzol Universal Reagent (Tiangen, China). Complementary DNA (cDNA) synthesis for circRNA and mRNA was performed with 1 μg of total RNA using the PrimerScript™ RT Reagent Kit (TaKaRa, Japan). *GAPDH* and *ACTB* were separately used as internal standard controls for circRNA and mRNA. cDNA was synthesized for miRNA with 3–4 μg of total RNA via miRNA First Strand cDNA Synthesis (Stem‐loop Method) (Sangon Biotech, China). *U6* was used as the internal standard control for miRNA. The circRNA, miRNA and mRNA were amplified and detected by SYBR Green PCR Kit (TaKaRa, Japan). The 2^−ΔΔCt^ method was used to calculate relative expression. Three experiments were conducted with three replicates each. Primer sequence information is listed in Table 2.

**TABLE 2:** Primer sequence in qRT‐PCR analysis.

Name		Sequence
CircPVT1	Forward	GGTTCCACCAGCGTTATTC
	Reverse	CAACTTCCTTTGGGTCTCC
*HAVCR2*	Forward	CTGCTGCTACTACTTACAAGGTC
	Reverse	GCAGGGCAGATAGGCATTCT
*SEMA3A*	Forward	ACCCAACTATCAATGGGTGCCTTA
	Reverse	AACACTGGATTGTACATGGCTGGA
*DSCC1*	Forward	CGTGGTGATAAAGACGAGCA
	Reverse	CCGGAGTTTTACAACCAGGA
*GAPDH*	Forward	GCACCGTCAAGGCTGAGAAC
	Reverse	GGATCTCGCTCCTGGAAGATG
*U6*	Forward	CTCGCTTCGGCAGCACA
	Reverse	AACGCTTCACGAATTTGCGT
*ACTB*	Forward	CATGTACGTTGCTATCCAGGC
	Reverse	CGCTCGGTGAGGATCTTCATG
miR‐490‐5p	Stem‐loop reverse transcription	GTCGTATCCAGTGCAGGGTCCGAGGTATTCGCACTGGATACGACACCCAC
	Forward	GCAGCCATGGATCTCCAG
	Reverse	GTGCAGGGTCCGAGGT
miR‐484	Stem‐loop reverse transcription	GTCGTATCCAGTGCAGGGTCCGAGGTATTCGCACTGGATACGACATCGGG
	Forward	TCAGGCTCAGTCCCCT
	Reverse	GTGCAGGGTCCGAGGT
miR‐24‐3p	Stem‐loop reverse transcription	GTCGTATCCAGTGCAGGGTCCGAGGTATTCGCACTGGATACGACCTGTTC
	Forward	GGCTCAGTTCAGCAGGA
	Reverse	GTGCAGGGTCCGAGGT
miR‐148b‐3p	Stem‐loop reverse transcription	GTCGTATCCAGTGCAGGGTCCGAGGTATTCGCACTGGATACGACACAAAG
	Forward	GTCAGTGCATCACAGAACT
	Reverse	GTGCAGGGTCCGAGGT
miR‐30d‐5p	Stem‐loop reverse transcription	GTCGTATCCAGTGCAGGGTCCGAGGTATTCGCACTGGATACGACCTTCCA
	Forward	GTGTAAACATCCCCGACTG
	Reverse	GTGCAGGGTCCGAGGT
miR‐30e‐5p	Stem‐loop reverse transcription	GTCGTATCCAGTGCAGGGTCCGAGGTATTCGCACTGGATACGACCTTCCA
	Forward	GCAGTGTAAACATCCTTGACT
	Reverse	GTGCAGGGTCCGAGGT

*Note*: All sequences are written in 5′–3′ direction.

### 2.4 Ribonuclease R (RNase R) treatment

To verify the circRNA characteristics, 2 μg of total RNA was incubated in 4 U RNase R (Geneseed, China) for 20 min at 37°C, qRT‐PCR was performed to detect circPVT1 and *GAPDH* expression.

### 2.5 Fluorescence in situ hybridization (FISH)

Cy3‐labelled circPVT1 probes (TTCTGGCCAAAAGATCAGGCCTCAAGCCCAGCTGAGCGCC) were synthesized by GenePharma, China. The probe signals were detected by the FISH Kit (GenePharma, China). A confocal microscope (Leica, Germany) was used to capture images.

### 2.6 Wound healing assay

After transfection, 1000 μL pipette tips were used to scratch HOS and U2OS cells cultured in six‐well plates (time 0 h). Phosphate buffered saline (PBS) was used to wash cells and serum‐free medium (SFM) was used to incubate cells for 24 h. Images were captured with 50× magnification using a microscope (Leica, Germany) at 0 and 24 h after injury.

### 2.7 Transwell migration and Matrigel invasion assays

The migration assay was conducted using a transwell chamber (8.0 μm pore polycarbonate membrane, 24 well format) (Corning, USA). The Matrigel invasion assay was conducted using a pre‐coated Matrigel Invasion chamber (8.0 μm pore polycarbonate membrane, 24 well format) (Corning, USA). After transfection, 600 μL medium with 10% FBS was added to the lower chamber, and an additional 200 μL SFM with cells (4 × 10^4^) were seeded in the upper chamber. The migrated and invaded cells after 48 h of incubation were immobilized, stained, imaged and at least five random fields were counted with a 200× magnification under a microscope (Leica, Germany).

### 2.8 Bioinformatics analysis

The downregulated miRNAs in OS were identified in GSE65071 and GSE28423 datasets from the Gene Expression Omnibus (GEO) database with *p* < 0.05 and |log_2_ fold change (FC)|>1. The upregulated mRNAs in OS were identified in GSE11416 and GSE33382 datasets with *p* < 0.05 and |log_2_ FC|>1. The target miRNAs of circPVT1 and their binding sites were predicted using CircBank (http://www.circbank.cn/index.html) and StarBase (http://starbase.sysu.edu.cn/). The target mRNAs of miR‐490‐5p and their binding sites were predicted using miRDB (https://mirdb.org/) and TargetScan (https://www.targetscan.org/vert_72/).

### 2.9 Western blot

Total cellular protein was extracted using enhanced radioimmunoprecipitation assay (RIPA) lysis buffer and phenylmethylsulphonyls fluoride (PMSF) reagent (Bioss, China). Then, Bicinchoninic Acid (BCA) Assay Kit (Biosharp, China) was used to quantify protein. Sodium dodecyl sulfate polyacrylamide gel electrophoresis (SDS‐PAGE) was performed for the separation of target proteins, which were then transferred to polyvinylidene difluoride (PVDF) membrane (Millipore, Germany). Transfer condition was 200 mA for 100 min. The membranes with ACTB or HAVCR2 proteins were separately incubated in primary anti‐ACTB (1:200, ab115777, Abcam, UK) and anti‐HAVCR2 (1:1000, A2516, ABclonal) antibodies at 4°C overnight. Next, horseradish peroxidase (HRP) labelled secondary antibody IgG (1:5000, #511203, Zen Bio, China) was used for incubation of the membranes at room temperature (RT) for 2 h. Finally, signals were detected using a chemiluminescence system (Bio‐Rad, USA) after adding the UltraSignal ECL substrate (4A Biotech, China).

### 2.10 Dual‐luciferase reporter assay

The reporter vectors (pmirGLO vectors with wildtype or mutant circPVT1 sequence and pmirGLO vectors with wildtype or mutant *HAVCR2* sequence) were synthesized by GenePharma, China. Co‐transfection of the reporter vectors and miR‐490‐5p mimics or NC into HEK‐293 T cells was conducted using Lipofectamine 3000 (Invitrogen, USA). The luciferase activity after 24 h of co‐transfection was detected using a multi‐mode microplate reader (BioTek, USA) after processing with Dual‐Luciferase Reporter Gene Assay Kit (GenePharma, China).

### 2.11 Statistical analyses

Statistical analyses were conducted using GraphPad Prism 9. The data were presented as mean ± standard deviation (SD) and Student's *t*‐test was used to detect statistical difference between two groups. Two‐tailed *p* < 0.05 were considered statistically significant.

## 3 RESULTS

### 3.1 CircPVT1 is highly expressed in OS cells

**FIGURE 1:**
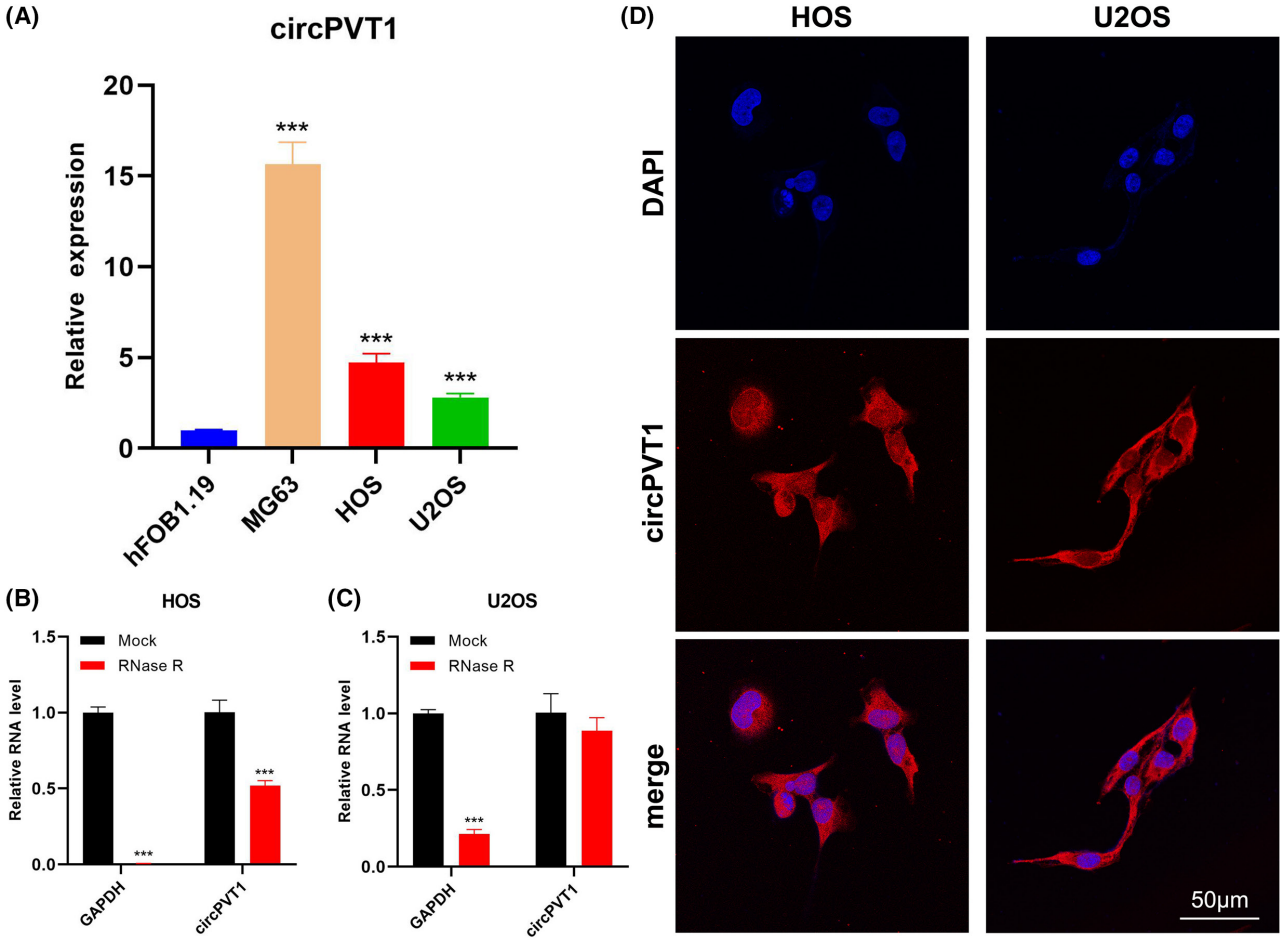
Expression of circPVT1 in OS cells. (A) Expression of circPVT1 in hFOB1.19 and OS cell lines (MG63, HOS, and U2OS) was measured by qRT‐PCR. Data were represented as mean ± SD (*n* = 3). (B, C) *GAPDH* and circPVT1 expression was measured in HOS and U2OS cells post RNase R treatment. (D) FISH indicated that circPVT1 localized mainly in the cytoplasm. Nuclei were visualized with DAPI (blue), and circPVT1 was visualized by probes labelled with Cy3 (red); scale bars, 50 μm. ***p < 0.001.

The qRT‐PCR results confirmed the upregulation of circPVT1 in OS cell lines, specifically MG63, HOS and U2OS, in comparison to the osteoblast cell line (hFOB1.19) (Figure 1A). Given the relatively low aggressiveness of MG63, subsequent experiments focused on migration and invasion analysis on the HOS and U2OS cells lines. Furthermore, the RNase R treatment assay validated the circRNA characteristics of circPVT1, with a notable reduction in the *GAPDH* level (Figure 1B,C). Subsequently, the circRNA FISH assay revealed that circPVT1 predominantly localized within the cytoplasm (Figure 1D). These collective findings strongly support that the upregulation of circPVT1 may function as a ceRNA in OS.

### 3.2 CircPVT1 promotes the migration and invasion of OS cells

**FIGURE 2:**
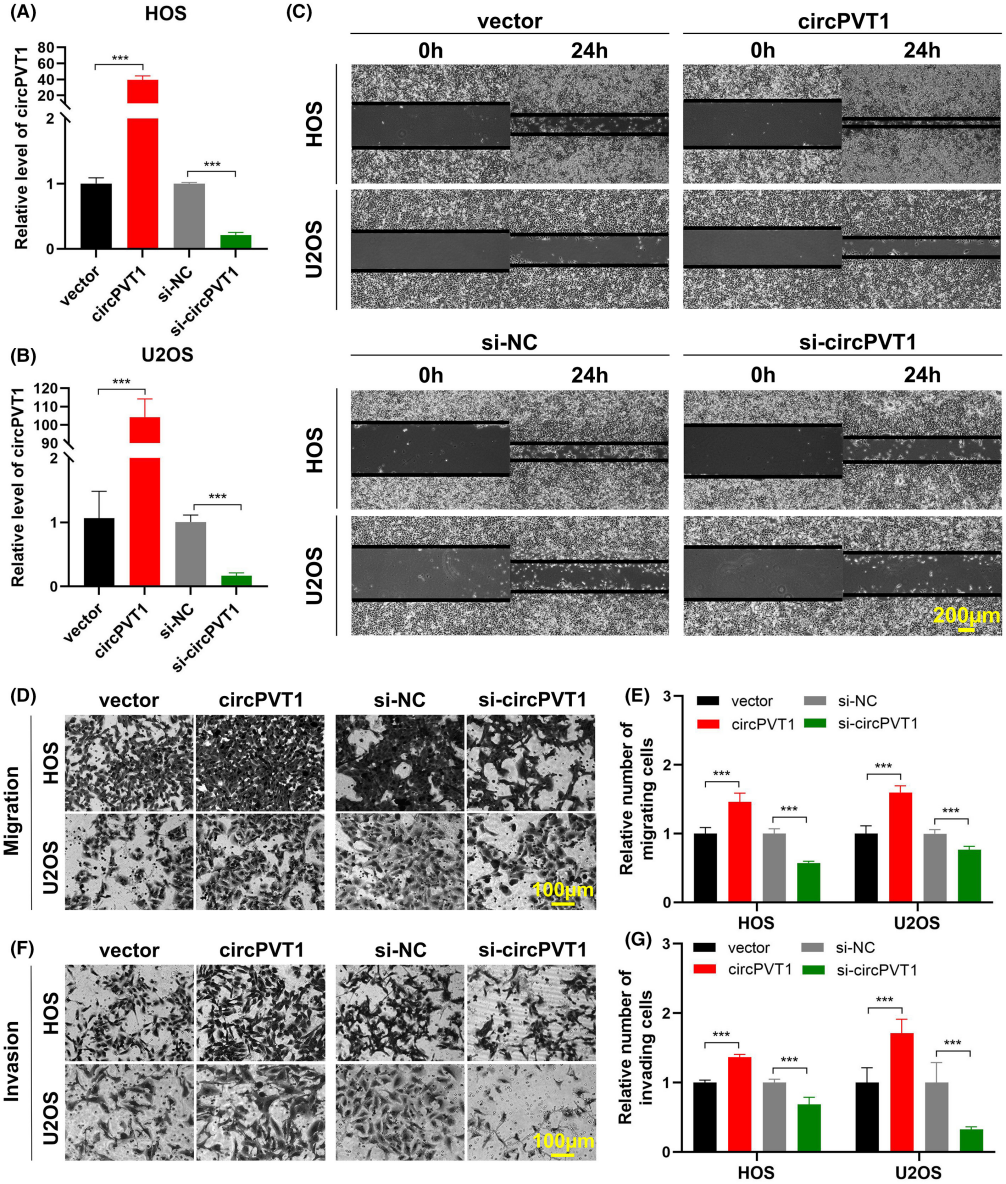
CircPVT1 facilitates OS cell migration and invasion. (A, B) qRT‐PCR detected CircPVT1 expression after transfection of the circPVT1 overexpression vector or siRNA. Data were represented as mean ± SD (*n* = 3). (C) Wound healing assay evaluated migration of HOS and U2OS cells following circPVT1 vector or siRNA transfection. Scale bar, 200 μm. (D–G) Transwell migration and Matrigel invasion assays assessed migration (D, E) and invasion (F, G) of HOS and U2OS cells following circPVT1 vector or siRNA transfection. Data were represented as mean ± SD (*n* = 5). Scale bar, 100 μm. ***p < 0.001.

To further explore the role of circPVT1 in OS cells, HOS and U2OS cells were transfected with the circPVT1 overexpression vector or siRNA, and the resulting alterations in circPVT1 expression were measured after 48 h (Figure 2A,B). The results demonstrated that circPVT1 overexpression notably enhanced the capacity of OS cell migration, evidenced by the wound healing and transwell migration assays, while circPVT1 inhibition had the opposite effect, diminishing the cells migratory protentional (Figure 2C–E). The Matrigel assay demonstrated an augmentation in cell invasion upon transfection with the circPVT1 overexpression vector, whereas a decrease in invasion was observed following transfection with the circPVT1 siRNA (Figure 2F,G).

### 3.3 CircPVT1 serves as a miR‐490‐5p sequester

**FIGURE 3:**
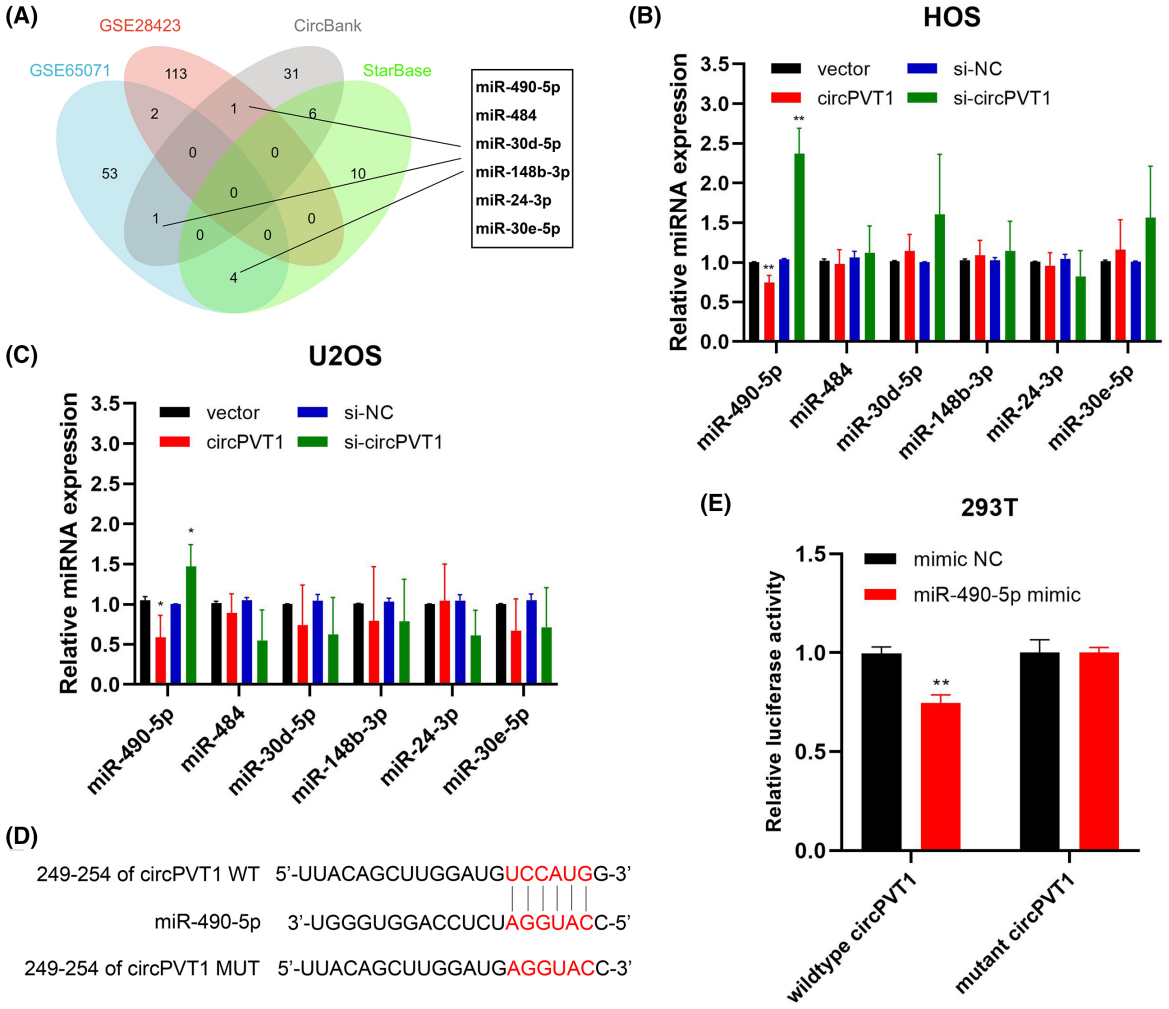
miR‐490‐5p is a direct target of circPVT1. (A) Downregulated miRNAs of OS were identified in GSE65071 and GSE28423 datasets with *p* < 0.05 and |log_2_ FC|>1 and potential miRNAs binding to circPVT1 were predicted using CircBank and StarBase. A Venn diagram showing the numbers of overlapping and non‐overlapping miRNAs in the databases. (B, C) The expression of six miRNAs was detected by qRT‐PCR in HOS and U2OS cells following the circPVT1 overexpression vector or siRNA transfection. Data were represented as mean ± SD (*n* = 3). (D) The predicted site of miR‐490‐5p for circPVT1. (E) Dual‐luciferase reporter assay detected the luciferase activity following wildtype or mutant circPVT1 luciferase reporter vector and miR‐490‐5p mimics or NC co‐transfection into HEK‐293 T cells. Data were represented as mean ± SD (*n* = 3). *p < 0.05, **p < 0.01.

To further explore the regulatory mechanism underlying the upregulated circPVT1 in OS progression, datasets GSE65071 and GSE28423 were selected to identify downregulated miRNAs in OS, applying a significance threshold of *p* < 0.05 and |log_2_ FC|>1. Additionally, we utilized CircBank and StarBase databases to predict potential target miRNAs associated with circPVT1. The bioinformatics analysis identified six downregulated miRNAs (miR‐484, miR‐24‐3p, miR‐148‐3p, miR‐30d‐5p, miR‐30e‐5p and miR‐490‐5p) that may be sequestered by circPVT1 (Figure 3A). qRT‐PCR was conducted to assess the expression level of six miRNAs in HOS and U2OS cells following circPVT1 overexpression or knockdown. As illustrated (Figure 3B,C), miR‐490‐5p emerged as the sole miRNA exhibiting upregulation in response to circPVT1 knockdown and downregulation upon circPVT1 overexpression in both HOS and U2OS cell lines. To investigate the binding interaction between circPVT1 and miR‐490‐5p, we conducted a dual‐luciferase reporter assay guided by the predicted binding site (Figure 3D). The results clearly demonstrated that the introduction of miR‐490‐5p mimics resulted in a reduction of luciferase activity within the wildtype circPVT1 group, while it had no discernable impact on the mutant circPVT1 group (Figure 3E). These findings collectively support that circPVT1 acts as a sequester for miR‐490‐5p.

### 3.4 miR‐490‐5p inhibits OS cell migration and invasion

**FIGURE 4:**
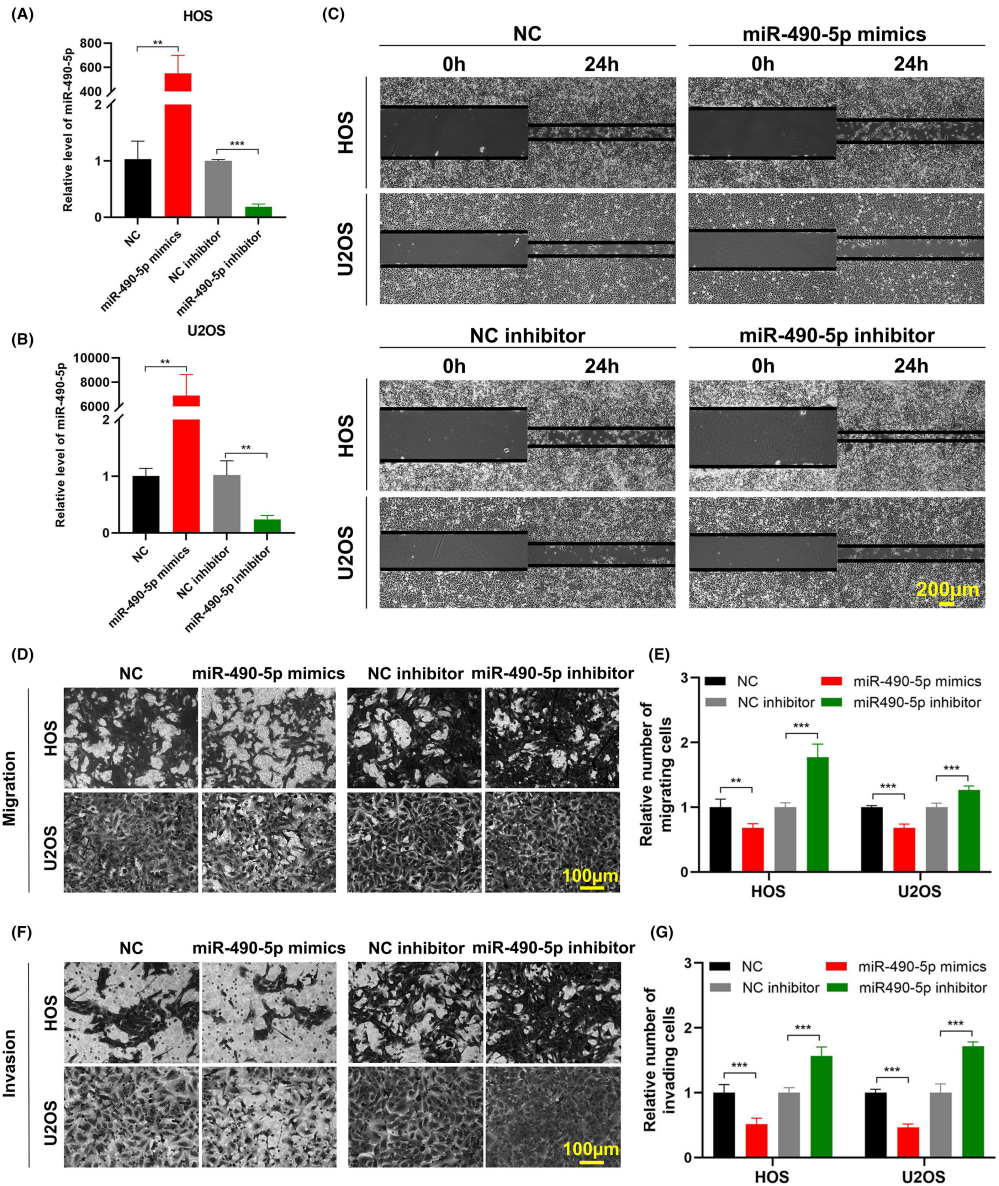
miR‐490‐5p inhibits OS cell migration and invasion. (A, B) miR‐490‐5p expression was evaluated by qRT‐PCR in HOS and U2OS cells following mimics or inhibitor transfection. Data were represented as mean ± SD (*n* = 3). (C) Wound healing assay assessed migration of HOS and U2OS cells following miR‐490‐5p mimics or inhibitor transfection. Scale bar, 200 μm. (D–G) Transwell migration and Matrigel invasion assays assessed migration (D, E) and invasion (F, G) of HOS and U2OS cells following miR‐490‐5p mimics or inhibitor transfection. Data were represented as mean ± SD (*n* = 5). Scale bar, 100 μm. **p < 0.01, ***p < 0.001.

While the inhibitory effects of miR‐490‐5p on cancer progression have been reported in various cancer types,^18^, ^19^, ^20^, ^21^ its specific impact on OS cells has not yet been explored. To elucidate the influence of miR‐490‐5p in the context of OS, we separately transfected miR‐490‐5p mimics and inhibitor into HOS and U2OS cells, to evaluate the transfection efficiency (Figure 4A,B). The results from both wound healing and transwell assays demonstrated that overexpression of miR‐490‐5p significantly inhibited the migration capacity of HOS and U2OS cells, while knockdown of miR‐490‐5p promoted cell migration (Figure 4C–E). Furthermore, the silencing of miR‐490‐5p promoted cell invasion, but the opposite effect was observed after overexpression (Figure 4F,G). In summary, these findings indicate that miR‐490‐5p exerts inhibitory effects on in vitro migration and invasion of OS cells.

### 3.5 *HAVCR2* is a target of miR‐490‐5p and considered as an oncogene in OS

**FIGURE 5:**
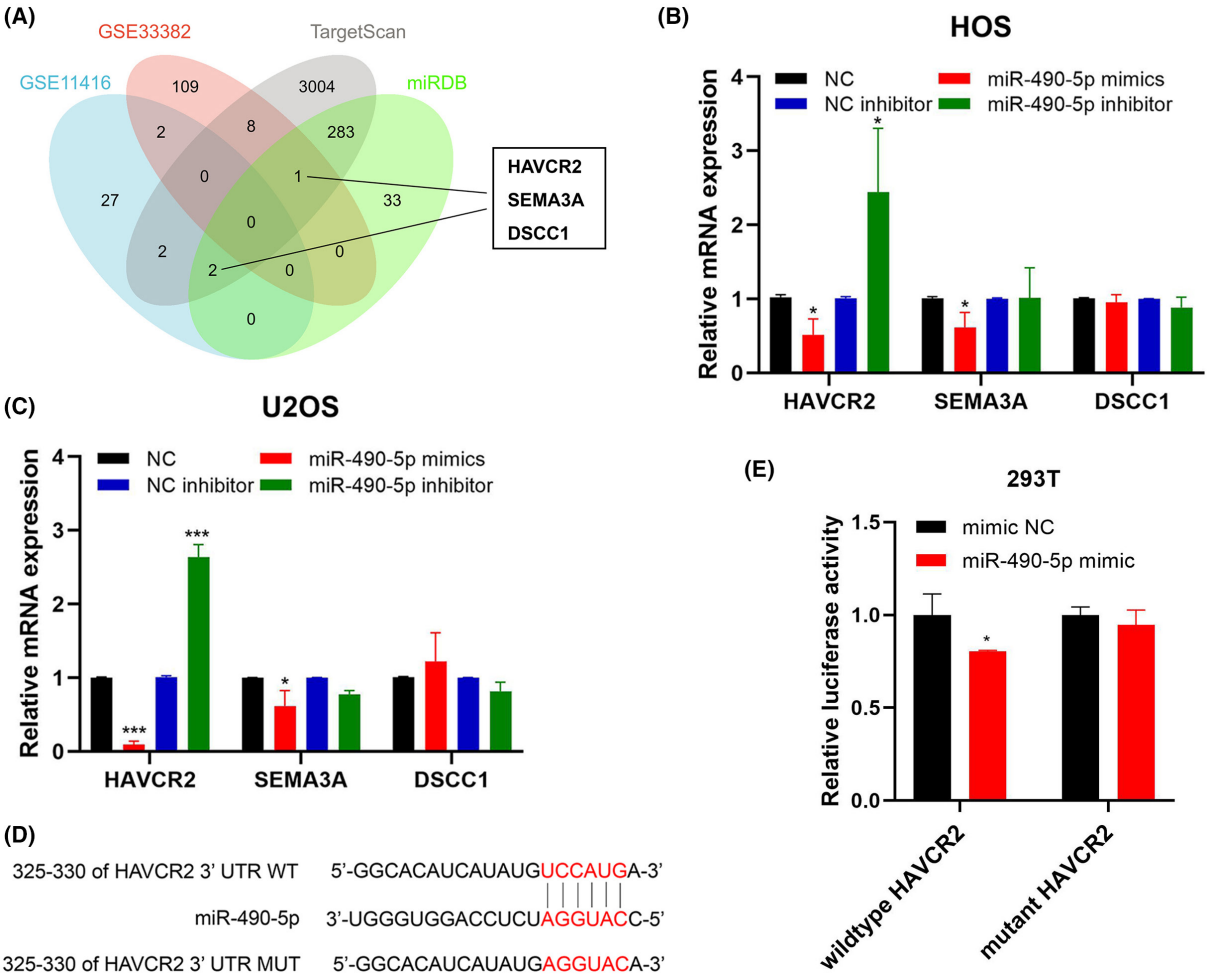
*HAVCR2* is a direct target of miR‐490‐5p. (A) Upregulated genes in OS were identified in GSE11416 or GSE33382 datasets with *p* < 0.05 and |log_2_ FC|>1 and potential target genes of miR‐490‐5p were predicted by TargetScan and miRDB. The Venn diagram displayed the overlapping genes. (B, C) The expression of three genes in HOS and U2OS following miR‐490‐5p mimics or inhibitor transfection were measured by qRT‐PCR. (D) The predicted binding site of *HAVCR2* 3′UTR and miR‐490‐5p. (E) The luciferase activity after co‐transfection of the wildtype or mutant *HAVCR2* 3′UTR luciferase reporter vector and miR‐490‐5p mimics or NC into HEK‐293 T cells were detected by dual‐luciferase reporter assay. Data were represented as mean ± SD (*n* = 3). *p < 0.05; ***p < 0.001.

**FIGURE 6:**
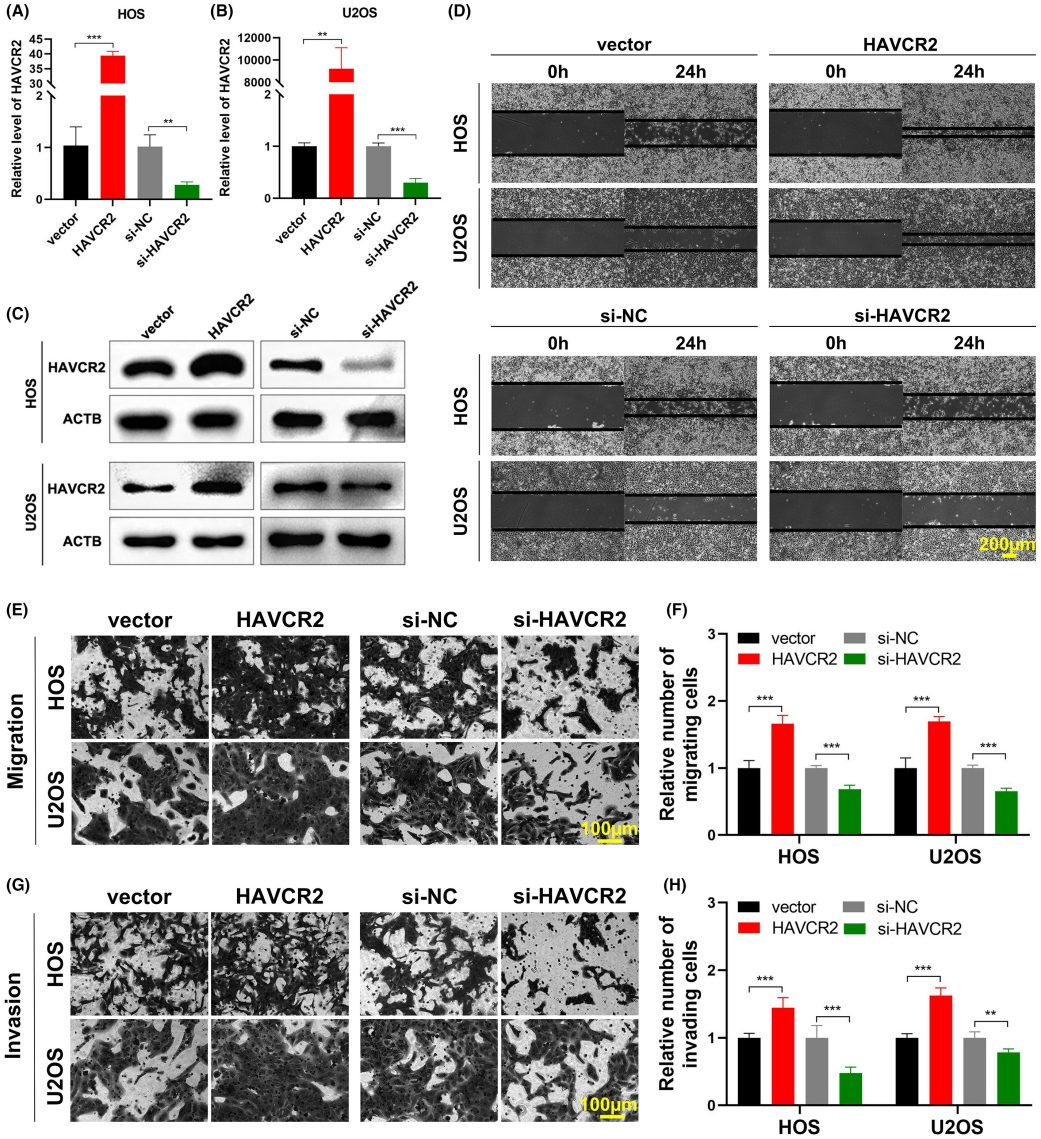
*HAVCR2* is an oncogene in OS cells. (A, B) The *HAVCR2* expression in HOS and U2OS following *HAVCR2* overexpression vector or siRNA transfection was detected by qRT‐PCR. Data were represented as mean ± SD (*n* = 3). (C) The HAVCR2 protein expression in HOS and U2OS cells following *HAVCR2* overexpression vector or siRNA transfection was detected by Western blot. (D) Wound healing assay assessed migration ability of HOS and U2OS cells following *HAVCR2* overexpression vector or siRNA transfection. Scale bar, 200 μm. (E–H) Transwell migration and Matrigel invasion assays assessed cell migration (E, F) and invasion (G, H) abilities of HOS and U2OS cells following *HAVCR2* overexpression vector or siRNA transfection. Data were represented as mean ± SD (*n* = 5). Scale bar, 100 μm. **p < 0.01; ***p < 0.001.

To evaluate the target genes under the regulation of miR‐490‐5p, the TargetScan and miRDB databases were employed and collectively 286 identified genes were shared between the two sources. Subsequently, we utilized the GSE11416 and GSE33382 datasets to pinpoint the overexpressed genes in OS that met the criteria of *p* < 0.05 and |log_2_ FC|>1. Three genes (*HAVCR2*, *SEMA3A* and *DSCC1*) exhibited substantial upregulation in OS and were concurrently predicted as potential targets of miR‐490‐5p (Figure 5A). Subsequently, the expression levels of all of three genes were assessed in HOS and U2OS cells following the transfection of miR‐490‐5p mimics or inhibitor. In HOS and U2OS cells, the expression of *HAVCR2* demonstrated a notable increase following miR‐490‐5p knockdown, while it decreased significantly after miR‐490‐5p overexpression (Figure 5B,C). Based on the predicted binding site for miR‐490‐5p in the 3′ untranslated region (UTR) of *HAVCR2*, a dual‐luciferase reporter assay was conducted to verify the direct interaction of miR‐490‐5p and *HAVCR2*. The reduction of luciferase activity was only found with the co‐transfection of the wild‐type *HAVCR2* reporter vector and miR‐490‐5p mimics (Figure 5D,E). The findings indicated that *HAVCR2* is likely to be a direct target of miR‐490‐5p. Subsequently, to identify the role of *HAVCR2* in HOS and U2OS cells, *HAVCR2* overexpression vector or siRNA were introduced into the OS cells, to evaluate their effectiveness through qRT‐PCR and Western blot (Figure 6A–C). Functionally, the findings from wound healing, transwell migration and Matrigel invasion assays indicated that *HAVCR2* knockdown led to a decrease in migration and invasion, whereas *HAVCR2* overexpression demonstrated contrasting results (Figure 6D–H), suggesting that *HAVCR2* has a role as an oncogene in OS.

### 3.6 CircPVT1 regulates OS migration and invasion through miR‐490‐5p/*HAVCR2* axis

**FIGURE 7:**
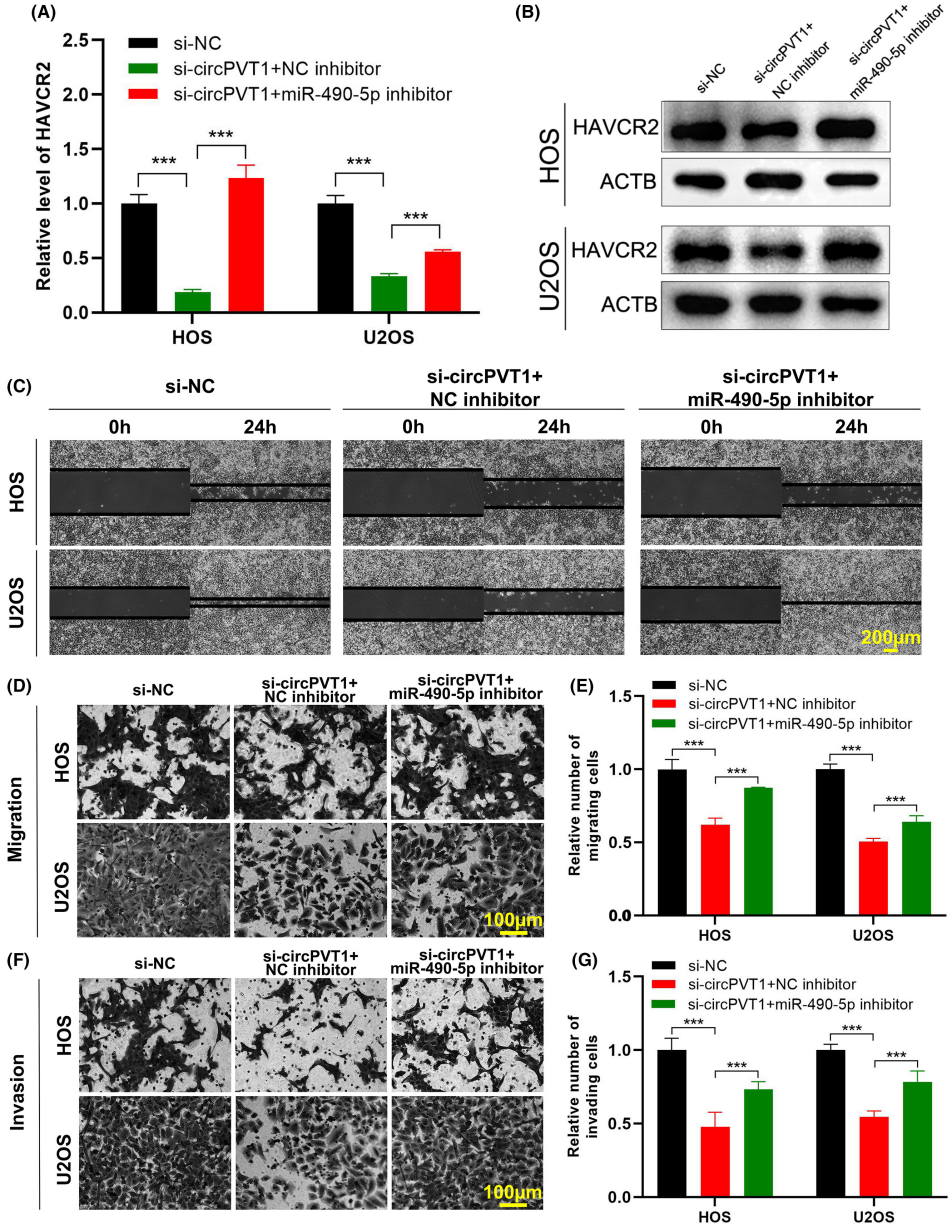
CircPVT1 regulates migration and invasion of OS cells through the miR‐490‐5p/*HAVCR2* axis. (A) qRT‐PCR evaluated the expression of *HAVCR2* after co‐transfection. Data were represented as mean ± SD (*n* = 3). (B) The HAVCR2 protein expression in HOS and U2OS cells after co‐transfection was evaluated by Western blot. (C) Wound healing assay accessed HOS and U2OS migration after co‐transfection. Scale bar, 200 μm. (D–G) Transwell migration and Matrigel invasion assays assessed cell migration (D, E) and invasion (F, G) abilities of OS cells after co‐transfection. Data were represented as mean ± SD (*n* = 5). Scale bar, 100 μm. ***p < 0.001.

Based on the theory of ceRNA, there might be a positive correlation between the expression of circPVT1 and *HAVCR2*. A relationship between circPVT1, miR‐490‐5p and *HAVCR2*, was further explored by co‐transfecting HOS and U2OS cells with circPVT1 siRNA and a miR‐490‐5p inhibitor. The results of qRT‐PCR and Western blot revealed a reduction in HAVCR2 levels following circPVT1 knockdown, which was subsequently rescued upon inhibition of miR‐490‐5p in HOS and U2OS cells (Figure 7A,B). These findings substantiated the regulatory role of circPVT1 on *HAVCR2* expression through miR‐490‐5p. Furthermore, the results from wound healing, transwell migration and Matrigel invasion assays revealed that silencing circPVT1 significantly suppressed cell migration and invasion. Interestingly, these inhibitory effects were mitigated by the inhibition of miR‐490‐5p in OS cells (Figure 7C–G). Considering these findings, it is plausible to conclude that circPVT1 likely regulates the migration and invasion capabilities of OS cells through miR‐490‐5p/*HAVCR2* axis.

## 4 DISCUSSION

The adjuvant chemotherapy has undeniably contributed to enhanced prognosis in OS patients since its implementation in 1980s, but there has been limited progress in survival rates. This is particularly the fact for metastatic and drug‐resistant cases.^4^, ^22^ Therefore, there exists a pressing imperative to identify potential therapeutic targets for refractory OS cases.

In recent years, the distinct attributes of circRNAs, characterized by their stable structures, inter‐species conservation and tissue specific and cell type‐specific expression, have attracted significant attention across various research domains, notably within the realm of cancer.^23^

Numerous experimental evidence has consistently underscored the dysregulation of circRNAs in OS, their involvements in various facets of tumorigenesis, migration, invasion, and resistance to chemotherapy. Consequently, circRNAs have emerged as promising candidates for novel therapeutic targets for OS therapy.^10^, ^11^, ^12^ Notably, circPVT1 has been previously recognized as an oncogene in OS, driving invasion and metastasis both in vitro and in vivo.^15^, ^16^ The current study builds upon these findings, further substantiating the role of circPVT1 in promoting the migration and invasion of OS cells.

While the precise regulatory mechanisms underlying circRNAs remain largely unclear, accumulating evidences suggest circRNAs, primarily those localized in the cytoplasm, function as ceRNAs by sequestering miRNAs.^24^ Previous studies have reported that circPVT1 exerts its influence on the biological behaviour of OS cells through the modulation of various miRNA axes, including miR‐526/*FOXC2*, miR‐26b‐5p/*CCNB1* and miR‐205‐5p/*c‐FLIP*.^14^, ^15^, ^16^

This current study showed that that circPVT1 primarily localized in the cytoplasm of HOS and U2OS cells by FISH. Furthermore, both qRT‐PCR and dual‐luciferase reporter assays provided compelling evidence of circPVT1 capacity to inhibit miR‐490‐5p through direct binding. Additionally, our investigations identified that circPVT1 knockdown exerted a suppressive effect on the migration and invasion of OS cells by inhibiting its target gene, *HAVCR2*. Remarkably, *HAVCR2* was identified as a target of miR‐490‐5p and was involved in promoting the progression of OS.

miR‐490‐5p is widely reported as a tumour suppressor, effectively targeting a range of genes to impede the progression of various cancers, including colon, pancreatic, kidney and liver malignancies.^18^, ^19^, ^20^, ^21^ Notably, miR‐490‐5p has also been reported to be sequestered by circRNAs in hepatocellular carcinoma and neuroblastoma.^25^, ^26^ Moreover, elevated miR‐490‐5p levels have been shown to inhibit cell migration and invasion in liver cancer.^20^, ^25^ However, no prior research has extensively explored the role of miR‐490‐5p in OS.

In this present study, we have discovered that miR‐490‐5p functions as a tumour suppressor in OS, inhibiting cell migration and invasion abilities. Furthermore, we identified *HAVCR2* as a direct target of miR‐490‐5p in OS, marking the first demonstration of miR‐490‐5p's capacity to inhibit *HAVCR2* expression in this context.

*HAVCR2*, also known as a T‐cell immunoglobulin and mucin‐domain‐3‐containing molecule3 (*TIM3*), belonging to the *TIM* gene family members of immunoregulatory proteins.^27^ Elevated HAVCR2 expression is a recurring phenomenon in cancer cells, it holds predictive power for aggressive disease progression and poor prognosis.^28^, ^29^ For instance, in gastric cancer, elevated HAVCR2 expression in tumour tissues is associated with poor prognosis.^29^ Similarly in cervical cancer, increased HAVCR2 expression is linked to advanced tumour grades, poor overall survival and metastatic potential.^30^

Furthermore, upregulated expression of HAVCR2 has been reported in both OS tissues and cell lines, with this overexpression correlating with poor prognosis in OS patients.^31^ In‐depth investigation into *HAVCR2* function, particularly in the MG63 cell line, has revealed that *HAVCR2* inhibition has a suppressive effect on tumorigenesis and metastasis, achieved through the inhibition of the NF‐Κb/Snail signalling pathway.^32^ In alignment with previous studies, our study provides an oncogenic role of *HAVCR2* in HOS and U2OS cells, identifying that *HAVCR2* upregulation promotes migration and invasion, while its inhibition yields the opposite effect. Nevertheless, the regulatory mechanism of *HAVCR2* on signalling pathways in the context of OS warrants further exploration.

HAVCR2 is widely recognized as an immune checkpoint expressed by immune cells, having gained prominence as a target for immune checkpoint target in recent years.^33^, ^34^ Moreover, the advantages of blocking HAVCR2 have been indicated in preclinical cancer models.^35^ In peripheral blood from OS patients, the expression of HAVCR2 is upregulated on CD8^+^ T‐cells, with higher levels associated with advanced tumour grade and metastasis.^36^, ^37^ Additionally, serum circPVT1 levels are elevated in OS patients compared to those with benign bone tumours and healthy controls.^17^

Exosomal circRNAs have been implicated in inducing dysfunction of CD8^+^ T‐cells, potentially influencing the effectiveness of immunotherapies in various cancers.^38^, ^39^ In light of our findings that circPVT1 can regulated *HAVCR2* via miR‐490‐5p, it is worth noting the possibility that circPVT1 may be assembled with serum‐derived exosomes, thereby potentially contributing to T cell dysfunction through the modulation of *HAVCR2*. This insight could hold significance in guiding decisions related to immunotherapeutic approaches for OS patients. Nevertheless, comprehensive exploration of the underlying mechanism involving circPVT1 in OS remains a subject requiring further research.

## 5 CONCLUSIONS

Our study unveiled the regulation of circPVT1 in OS cell lines and its role in promoting the migration and invasion of OS cells. This effect was achieved through the regulation of *HAVCR2* via miR‐490‐5p. These results provide a link between circPVT1, miR‐490‐5p, *HAVCR2* and the progression of OS, shedding light in the potential of this axis as a therapeutic target for the treatment of OS.

## AUTHOR CONTRIBUTIONS

**Chunbin Zhou:** Data curation (lead); formal analysis (lead); methodology (equal); visualization (lead); writing – original draft (lead). **Lois Balmer:** Methodology (supporting); project administration (supporting); supervision (supporting); writing – review and editing (lead). **Manshu Song:** Project administration (supporting); supervision (supporting); writing – review and editing (supporting). **Kezhou Wu:** Data curation (supporting); methodology (equal); writing – review and editing (supporting). **Wei Wang:** Project administration (supporting); supervision (lead); validation (equal); writing – review and editing (supporting). **Hu Wang:** Project administration (lead); resources (lead); supervision (supporting); validation (equal); writing – review and editing (supporting).

## FUNDING INFORMATION

This work was supported by Provincial Science and Technology Special Fund of Guangdong, China (grant number 210712096871702).

## CONFLICT OF INTEREST STATEMENT

The authors confirm that there are no conflicts of interest.

## Data Availability

The data that support the findings of this study are available from the corresponding author upon reasonable request.
